# Galectin-8 Ameliorates Murine Autoimmune Ocular Pathology and Promotes a Regulatory T Cell Response

**DOI:** 10.1371/journal.pone.0130772

**Published:** 2015-06-30

**Authors:** James F. Sampson, Eiichi Hasegawa, Lama Mulki, Amol Suryawanshi, Shuhong Jiang, Wei-Sheng Chen, Gabriel A. Rabinovich, Kip M. Connor, Noorjahan Panjwani

**Affiliations:** 1 Program in Immunology, Sackler School of Graduate Biomedical Sciences, Tufts University, Boston, MA, United States of America; 2 Angiogenesis Laboratory, Department of Ophthalmology, Harvard Medical School, Massachusetts Eye & Ear Infirmary, Boston, MA, United States of America; 3 New England Eye Center/Department of Ophthalmology, Tufts University School of Medicine, Boston, MA, United States of America; 4 Laboratory for Nutrition and Vision Research, JM USDA HNRCA at Tufts University, Boston, MA, United States of America; 5 Ophthalmology Department, People's Hospital of Inner Mongolia, Hohhot, China; 6 Cell, Molecular, & Developmental Biology, Sackler School of Graduate Biomedical Sciences, Tufts University, Boston, MA, United States of America; 7 Laboratorio de Inmunopatología, Instituto de Biología y Medicina Experimental (IBYME), Consejo Nacional de Investigaciones Científicas y Técnicas (CONICET), C1428 Buenos Aires, Argentina; Wayne State University, UNITED STATES

## Abstract

Galectins have emerged as potent immunoregulatory agents that control chronic inflammation through distinct mechanisms. Here, we report that treatment with Galectin-8 (Gal-8), a tandem-repeat member of the galectin family, reduces retinal pathology and prevents photoreceptor cell damage in a murine model of experimental autoimmune uveitis. Gal-8 treatment increased the number of regulatory T cells (Treg) in both the draining lymph node (dLN) and the inflamed retina. Moreover, a greater percentage of Treg cells in the dLN and retina of Gal-8 treated animals expressed the inhibitory coreceptor cytotoxic T lymphocyte antigen (CTLA)-4, the immunosuppressive cytokine IL-10, and the tissue-homing integrin CD103. Treg cells in the retina of Gal-8-treated mice were primarily inducible Treg cells that lack the expression of neuropilin-1. In addition, Gal-8 treatment blunted production of inflammatory cytokines by retinal T helper type (T_H_) 1 and T_H_17 cells. The effect of Gal-8 on T cell differentiation and/or function was specific for tissues undergoing an active immune response, as Gal-8 treatment had no effect on T cell populations in the spleen. Given the need for rational therapies for managing human uveitis, Gal-8 emerges as an attractive therapeutic candidate not only for treating retinal autoimmune diseases, but also for other T_H_1- and T_H_17-mediated inflammatory disorders.

## Introduction

Autoimmune uveitis is a spectrum of inflammatory diseases that can affect any part of the eye, and collectively results in 10–20% of all cases of blindness in the United States [1]. Patients with autoimmune uveitis display strong T helper type (T_H_)1 and T_H_17 responses [2],[3], and are relatively deficient in regulatory T cells (Treg cells) [4]. Surface expression of the inhibitory coreceptor cytotoxic T lymphocyte antigen-4 (CTLA-4) on Treg cells is higher in uveitis patients who respond well to treatment than on Treg cells from patients with active disease [4]. The immunopathology of uveitis has been extensively studied using rodent models of experimental autoimmune uveitis (EAU), which faithfully recapitulate aspects of human uveitis pathology, including immune cell pathophysiology. These studies have shown that depletion of Treg cells during active disease significantly increases EAU severity and favors the presence of T_H_1 and T_H_17 cells in draining lymph nodes (dLN) [5]. Conversely, Treg cell infusion before the onset of EAU ameliorates pathology [6]. Previous work has shown that skewing the T cell response towards T_H_2 and Treg and away from T_H_1 and T_H_17 suppresses EAU [7].

Due to the key role of Treg cells in preventing autoimmunity, there is intense interest in manipulating the signals responsible for generating and maintaining these cells. Although the generation and regulation of Treg cells is a complex and incompletely understood process, it is known that Treg cell development in the periphery depends on interleukin-2 (IL-2) and TGFβ signaling [8]. Recent studies have provided evidence that members of the galectin family also have the potential to modulate the generation and stability of Treg cells [7],[9–13]. Galectins constitute a family of animal lectins characterized by their affinity for β-galactoside-containing glycans. Galectins play an important role in many biological processes including, but not limited to, immune regulation, host-pathogen interactions, angiogenesis, and fibrosis [14–17]. In recent years, the ability of galectins to regulate the immune system has attracted much interest based on accumulating evidence implicating members of the galectin family as a novel class of modulators of innate and adaptive immune functions, and their potential as therapeutic agents for autoimmune disorders. Galectin-9 (Gal-9) has been shown to significantly reduce pathology of experimental autoimmune encephalomyelitis (EAE), a mouse model of multiple sclerosis [18], whereas Gal-1 prevents ocular pathology in EAU [7] as well as EAE [19]. Gal-3 inhibits Treg cell differentiation and function [20], whereas Gal-1 and -9 enhance the frequency and immunosuppressive capacity of Treg cells [12],[21]. Gal-8 is a tandem-repeat type member of the galectin family, with two structurally distinct carbohydrate recognition domains (CRDs). The N-terminal CRD preferentially binds to α2,3-sialylated glycans, a unique specificity among galectins [22]. Although the expression of Gal-8 is markedly increased in response to inflammation (Chen, et al. in preparation), its role in the regulation of the immune system is poorly understood, and nothing is known about the role of Gal-8 in autoimmune diseases such as uveitis.

We demonstrate here that Gal-8 treatment reduces retinal pathology and photoreceptor cell damage in the mouse model of EAU, and that reduction in retinal pathology is associated with a concomitant increase in the anti-inflammatory Treg cell response in the dLN and retina, and a decrease in T_H_1 and T_H_17 cytokine production locally in the retina. We further show that a higher percentage of Treg cells from Gal-8-treated mice express the inhibitory coreceptor, CTLA-4, the immunosuppressive cytokine IL-10, and the tissue-homing integrin CD103, as compared to Treg cells from vehicle-treated mice. Thus, the inhibitory effect of Gal-8 on EAU appears to be a result of selectively modulating the immune response in the eye.

## Materials and Methods

### Ethics statement

All animal procedures were approved by the IACUC committees of Tufts University (#B2013-159) and Harvard Medical School (#10-032A). Mice were anesthetized with avertin prior to ocular imaging. Prior to euthanasia by cervical dislocation, mice were administered ketamine and xylazine.

### Mice

C57BL/6J (B6) mice were obtained from the Jackson Laboratory and maintained in animal facilities approved by the Association for Assessment and Accreditation of Laboratory Animal Care (AAALAC) at Tufts University (IACUC #B2013-159) and Harvard Medical School (IACUC #10-032A). Unlike C57BL/6N mice, B6J mice do not have the *Rd8* mutation that causes retinal pathology [23]. This was verified by genotyping. All animal study protocols conformed to the Association for Research in Vision and Ophthalmology resolution on the use of Animals in Vision Research and the recommendations of the National Institutes of Health Guide for the Care and Use of Laboratory Animals.

### Reagents, Antigens (Ags), and Monoclonal Antibodies (mAbs)

CFA was purchased from Sigma, and *B*. *pertussis* toxin was purchased from Invitrogen. Human interphotoreceptor retinoid-binding protein (IRBP) peptide_1–20_ (GPTHLFQPSLVLDMAKVLLD) was purchased from Biomatik. The following antibodies were purchased from eBioscience, as conjugated to FITC, PE, or allophycocyanin: IFNγ XMG1.2), IL-17A (eBio17B7), Forkhead box P3 (Foxp3, FJK-16S), CTLA-4 (UC10-4B9), IL-10 (JES5-16E3), neuropilin-1 (3DS304M), and isotype controls. The following antibodies were purchased from BD, as conjugated to FITC, PE, or allophycocyanin: CD4 (RM4-5), CD103 (M290), IL-4 (11B11), and isotype controls.

### Preparation of recombinant human glutathione S-transferase tagged Gal-8

Recombinant human glutathione *S*-transferase (GST) tagged Gal-8 was produced and purified as previously described [24]. Briefly, lysates of bacteria expressing GST-Gal-8 were chromatographed on a β-lactose-conjugated Sepharose column, and bound to an affinity matrix (EY Labs; 1 ml bed volume). GST-Gal-8 was eluted from the column with lactose. Fractions containing the lectin were dialyzed against PBS containing 2% glycerol and 4 mM β-mercaptoethanol and stored at −80°C.

### EAU induction and treatment with Gal-8

EAU was induced in eight week old B6 female mice as previously described [25],[26]. Briefly, B6 mice were immunized s.c. in the scruff, femur, and foot pad with a total of 200 μg of IRBP peptide_1–20_ emulsified in complete Freund’s adjuvant (CFA, H37Ra, Difco Laboratories). Mice were simultaneously injected i.p. with 100 μl (1.5 μg) of *B*. *pertussis* toxin. On days 2, 4, 6, 14, 16, 18, and 22 post immunization (p.i.), mice were injected i.p. with 50 μg Gal-8 diluted in 100 μl PBS or an equal volume of buffer control (vehicle). Gal-8 treatment began on day 2 to ensure that the lectin was present during T cell activation [7], and we selected 50 μg per mouse as has been previously used for other galectins [7].

### Imaging mouse fundus and grading system

Mice were anesthetized with an i.p. injection of avertin (Sigma) diluted in saline. Pupils were dilated with one drop of a solution of 0.5% (w/v) tropicamide and 5% (w/v) phenylephrine (MEEI pharmacy). Goniovisc (2.5%, HUB pharmaceuticals) was used as a corneal medium during imaging. A Micron III Retinal Imaging Microscope and StreamPix software (Phoenix Research Labs) were used to image the fundus. Scoring was as reported previously [7],[26]. Briefly, retinal infiltrates, optic disc changes, vascularity, and structural damage were scored on a scale of 0–4. Infiltrates: 1 = 1–4 small lesions or 1 linear lesion, 2 = 5–10 small lesions or 2–3 linear lesions, 3 = >10 small lesions or >3 linear lesions, 4 = confluent linear lesions. Optic disc: 1 = minimal inflammation, 2 = mild inflammation, 3 = moderate inflammation, 4 = severe inflammation. Retinal vessels: 1 = engorged vessels, no cuffing, 2 = engorged vessels, 1–4 mild cuffs, 3 = >4 mild cuffs or 1–3 moderate cuffs, 4 = >3 moderate cuffs, >1 severe cuff. Structural damage: 1 = lesions or atrophy on <1/4 of retina, 2 = lesions or atrophy on 1/4-3/4 of retina, 3 = pan retinal atrophy with multiple small scars or <3 linear scars, 4 = pan retinal atrophy with >3 linear or confluent scars. Clinical score was calculated by averaging the score for each of the four criteria.

### Determination of photoreceptor pathology

Mice were anesthetized with avertin to prevent movement and pupils were dilated with tropicamide and phenylephrine as above. Mice were then placed on a custom, free-rotating platform while focusing on the retina. Lesions were first identified by fundus imaging, then spectral domain optical coherence tomography (OCT) images were acquired using a Micron III Retinal Imaging Microscope with an OCT attachment (Phoenix Research Labs). Ten sequential images were averaged to yield the presented composite images.

### Histopathology

To confirm OCT and fundoscopy observations, eyes were enucleated 24 d p.i. and processed for histopathology. Briefly, eyes were fixed in Davidson’s fixative (32% ethanol, 2.2% buffered formalin, 11% glacial acetic acid) for 24 h, embedded in paraffin, and 5 μm sections were stained with standard eosin and hematoxylin [27]. Presence or absence of disease was scored in a blinded fashion by two independent observers examining 10 sections of each eye at different depths. Severity of EAU was assessed on a scale of 0–4 as described previously [28].

### Isolation of eye-infiltrating cells and preparation of retinal supernatant

Uveitic eyes were collected from 5 mice per group, and trimmed along the limbus for removal of external tissue, as described previously [29]. Pooled retinal tissue was minced with scissors in cRPMI containing complete protease inhibitor cocktail (Roche), and dissociated with vigorous pipetting. After centrifugation, clarified supernatant (retinal extract) was used for ELISA. Pellets were digested with 60 U/ml Liberase (Roche) for 1 h at 37°C, washed with cRPMI, and passed through a 70 μm cell strainer to remove debris, and the cells were used for intracellular staining as below.

### Flow cytometry

Cells were stimulated with 50 ng/ml PMA and 500 ng/ml ionomycin (both Sigma) for 4 h in the presence of GolgiStop (BD) before intracellular cytokine staining. Cells were first stained for the surface markers CD4, neuropilin-1, CTLA-4, and/or CD103, and then fixed with Cytofix Buffer (BD) for cytokine staining or Fixation/Permeabilization Buffer (eBioscience) for nuclear staining of Foxp3. Cells were stained with appropriately diluted fluorophore-labeled antibodies against intracellular targets (Foxp3, IL-4, IFNγ, IL-17A) in buffer containing 0.5% saponin (Sigma). Flow cytometry was performed on a FACS Calibur (BD), and data were analyzed with FlowJo software (Tree Star). Gates were set based on appropriate isotype controls. Where indicated, Fold Change represents percentage or number of marker positive cells in gated population as compared to control. On overlayed histograms, % of Max represents the total number of cells at a given fluorescence intensity compared to the maximum number of cells at any intensity for a given sample.

### Cytokine ELISA

Retinal extracts were assayed directly. Supernatants from dLN cell cultures were collected 48 h after activation with 30 μg/ml IRBP_1-20_ and secreted cytokines in the supernatants were measured by ELISA using kits with purified capture and biotinylated detection antibodies against IFNγ, IL-4, IL-17A, and IL-10 (eBioscience). Where indicated, fold change represents concentration of cytokine as compared to vehicle treatment.

### Statistical analysis

Statistical analysis was performed using two-way unpaired Student’s *t* test as indicated. Analysis of uveitis scores and histopathology was performed using the Mann-Whitney *U* nonparametric test. P-values were considered statistically significant at p < 0.05 (*), p < 0.01 (**), p < 0.001 (***), p < 0.0001 (****).

## Results

### Galectin-8 attenuates retinal pathology in experimental autoimmune uveitis

**Fig 1:**
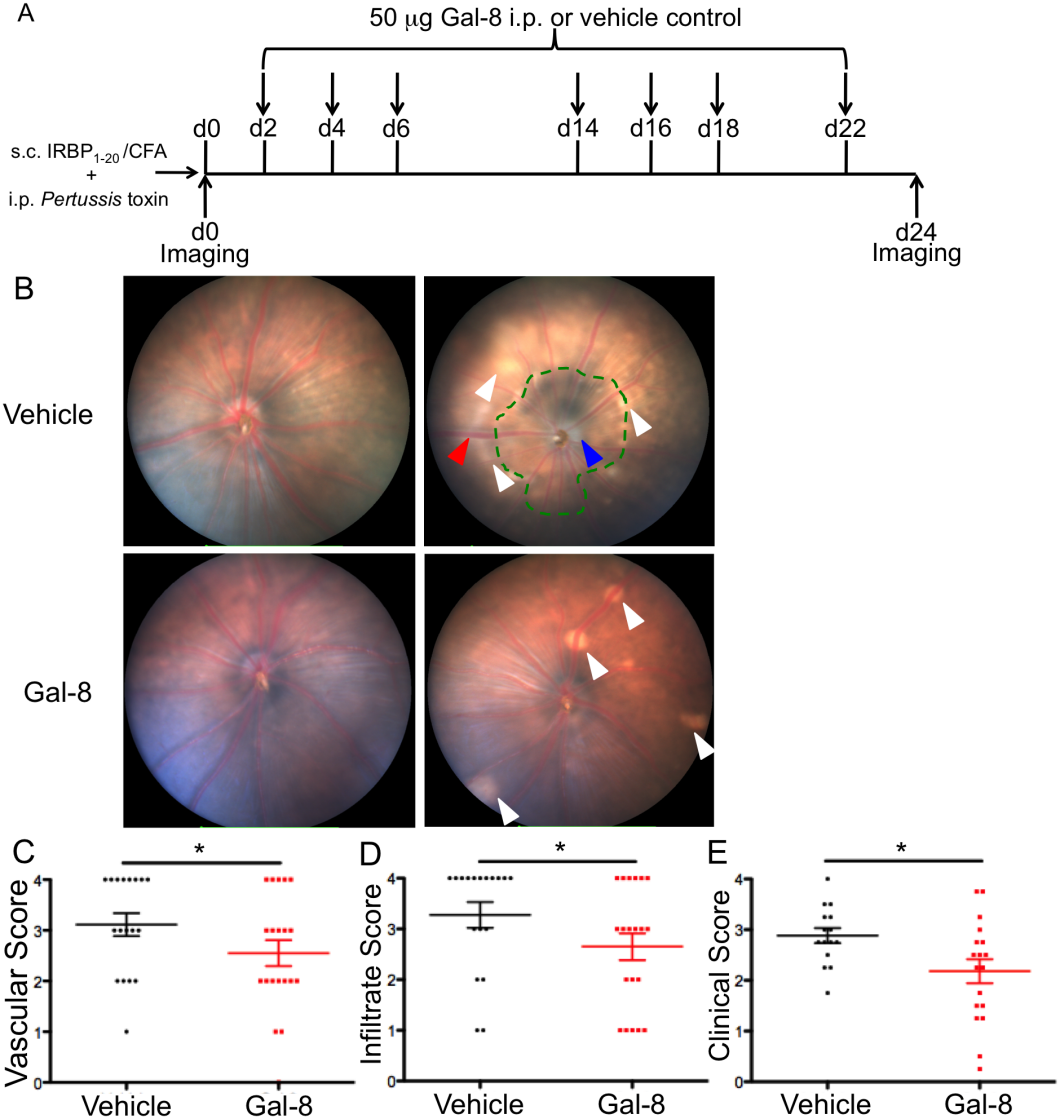
Galectin-8 ameliorates EAU pathology. (A) B6 mice were immunized with IRBP peptide (1–20) to induce uveitis. Groups of at least 5 mice each were injected with vehicle or Gal-8 and imaged as indicated. (B) Representative fundus images of vehicle- and Gal-8-treated mice at day 0 and 24. Blue arrowhead: optic disc inflammation; white arrowhead: immune cell infiltrates; red arrowhead: engorged vasculature; green dotted line: retinal atrophy. Retinal vascular changes (C), lymphocyte infiltration to the retina (D), and overall uveitis clinical score (E) were determined for the most severe eye of each mouse and averaged across three independent experiments with at least five vehicle- and five Gal-8-treated mice each. Error bars are SEM from three independent experiments with a total of 18 vehicle- and 20 Gal-8-treated mice. P values were determined by Mann-Whitney *U* test. *, p < 0.05.

EAU is an ocular autoimmune disease driven by T_H_1 and T_H_17 cells and ameliorated by Treg cells [6],[29]. To determine whether Gal-8 has anti-inflammatory activity *in vivo*, EAU was induced in two groups of mice by subcutaneous immunization with IRBP_1-20_ emulsified in CFA, along with simultaneous i.p. injections of *pertussis* toxin [25],[26]. One group of mice received 7 doses of Gal-8 (50 μg in 100 μl) on the indicated days (Fig 1A). The control group of mice received i.p. injections of vehicle alone. No notable side effects or behavioral changes were detected after Gal-8 treatment. Eyes were imaged on the day of immunization and 24 days later, at the termination of the experiment (Fig 1A). Representative fundus (inner lining of the eye) images of the central retina of mice treated with either vehicle or recombinant Gal-8 on day 24 p.i. are shown in Fig 1B. The images were scored for vascular changes, immune cell infiltration, optic disc inflammation, and structural damage, and the four scores were averaged to give an overall clinical score. Inflammatory lesions were more prevalent in retinas from vehicle-treated compared to Gal-8-treated mice (Fig 1B, white arrowheads). Vehicle-treated mice also exhibited large, discolored regions of atrophy in the central retina (Fig 1B, green outline). In contrast, little atrophy was seen in the retinas of Gal-8-treated mice. Also, Gal-8 treatment substantially reduced optic disc inflammation detected in the vehicle-treated mice (Fig 1B, blue arrowhead). Overall, compared to vehicle-treated mice, retinas of Gal-8-treated mice had significantly reduced vascular swelling, cuffing and immune cell infiltration (Fig 1C and 1D), reduced inflammation of the optic disc and structural changes, and reduced overall clinical score (Fig 1E). Thus, treatment with Gal-8 reduces EAU pathology.

**Fig 2:**
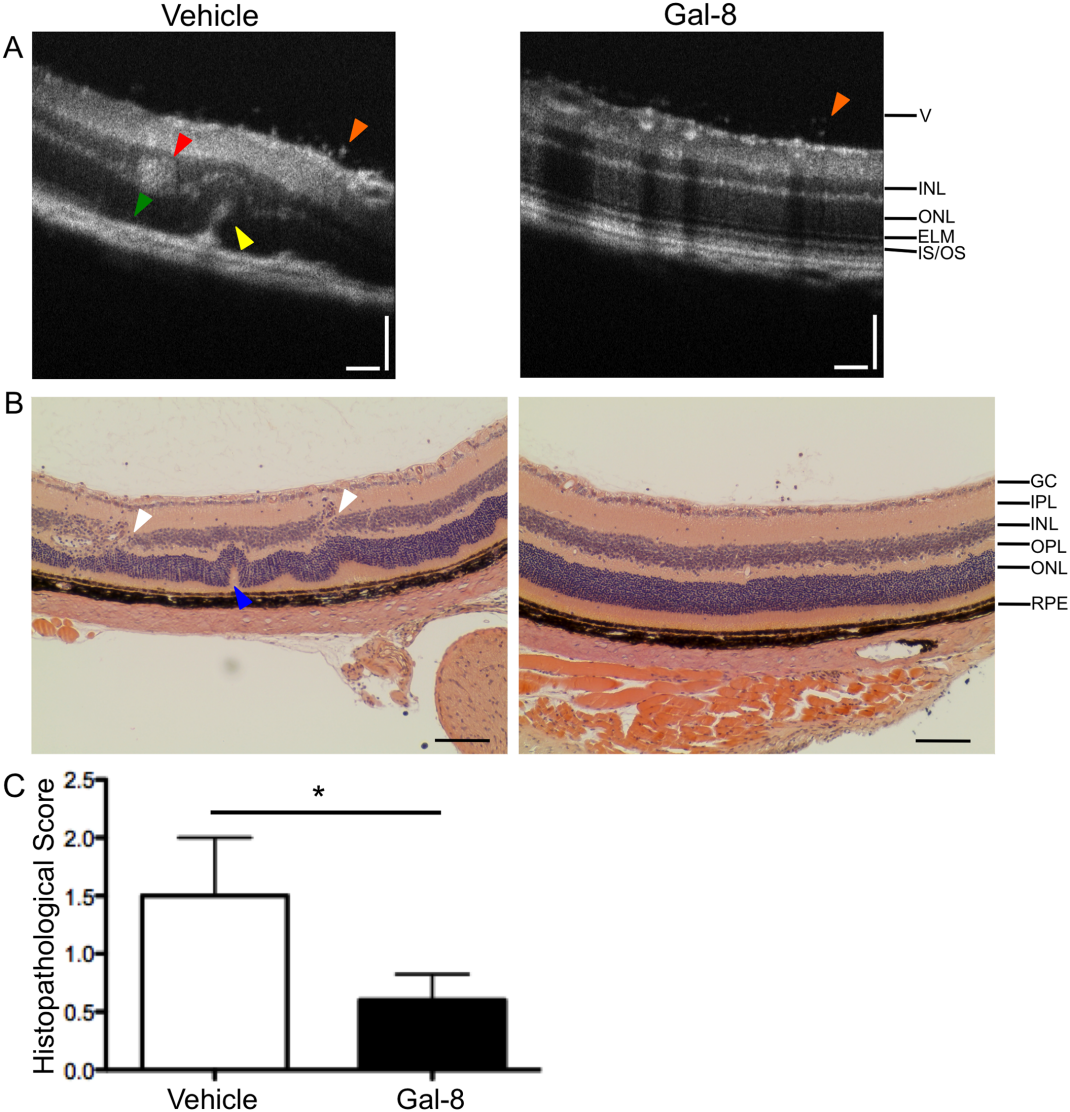
Galectin-8 treatment reduces damage to the neural retina. B6 mice were immunized with IRBP peptide and injected with vehicle or Gal-8 as shown in Fig 1. (A) Representative OCT images of retinal lesions at day 24 p.i. Composite images are average of ten adjacent images. Red arrowhead: inflammatory debris; green arrowhead: degradation of the ELM and IS/OS; yellow arrowhead: disruption of the middle retina by hyper-reflective material; orange arrowhead: vitreous infiltrate. Vertical and horizontal scale bars, 50 μm. Images are representative from three independent experiments with a total of 18 vehicle- and 20 Gal-8-treated mice (B) Representative histopathology of damage to the retina in vehicle- and Gal-8-treated mice at day 24 p.i. White arrowhead: disruption of the INL; blue arrowhead: retinal folding. Scale bar, 50 μm. Images are representative of two independent experiments with 5 vehicle- and 5 Gal-8-treated eyes. V: vitreous; GC: ganglion cell layer; IPL: inner plexiform layer; INL: inner nuclear layer; OPL: outer plexiform layer; ONL: outer nuclear layer; ELM: external limiting membrane; IS/OS: Inner segment/ outer segment junction; RPE: retinal pigment epithelium. (C) Histopathology scores of vehicle- and Gal-8-treated eyes. At least 10 slides from different depths were scored and averaged to give a value for each eye. Error bars are mean ± SEM from two independent experiments with 5 vehicle- and 5 Gal-8-treated eyes. P values were determined by Mann-Whitney *U* test. *, p < 0.05.

To determine whether treatment with Gal-8 affected damage to the neural retina during EAU, 24 days p.i., retinal lesions were identified by fundus imaging, and then imaged by OCT, a commonly used clinical technique that allows cross sectional imaging of live tissue. Vehicle-treated mice contained infiltration of cells into the vitreous (V, orange arrowhead), as well as hyper-reflective material in the outer nuclear layer (ONL, Fig 2A, yellow arrowhead), possibly indicative of active inflammation, as expected. Additionally, these mice had inflammatory debris in the inner nuclear layer (INL, Fig 2A, red arrowhead) and degradation of the external limiting membrane (ELM) and inner segment/outer segment (IS/OS) junction (Fig 2A, green arrowhead), suggesting photoreceptor cell damage. Lesions in Gal-8-treated mice primarily had reduced vitreous infiltrates, and little to no disturbance of the photoreceptors (Fig 2A). These results were confirmed by histopathology of eyes at 24 days p.i. (Fig 2B). In vehicle-treated mice, we observed ONL folding, granulomatous infiltration, and regions of discontinuity of the INL (Fig 2B). In contrast, Gal-8-treated mice had significantly reduced pathology scores (Fig 2B), and primarily had sparse infiltrating retinal cells without characteristics of severe inflammation or damage (Fig 2C), in agreement with the fundoscopy and OCT analysis.

### Gal-8 treatment *in vivo* enhances the Treg cell response in draining lymph nodes

**Fig 3:**
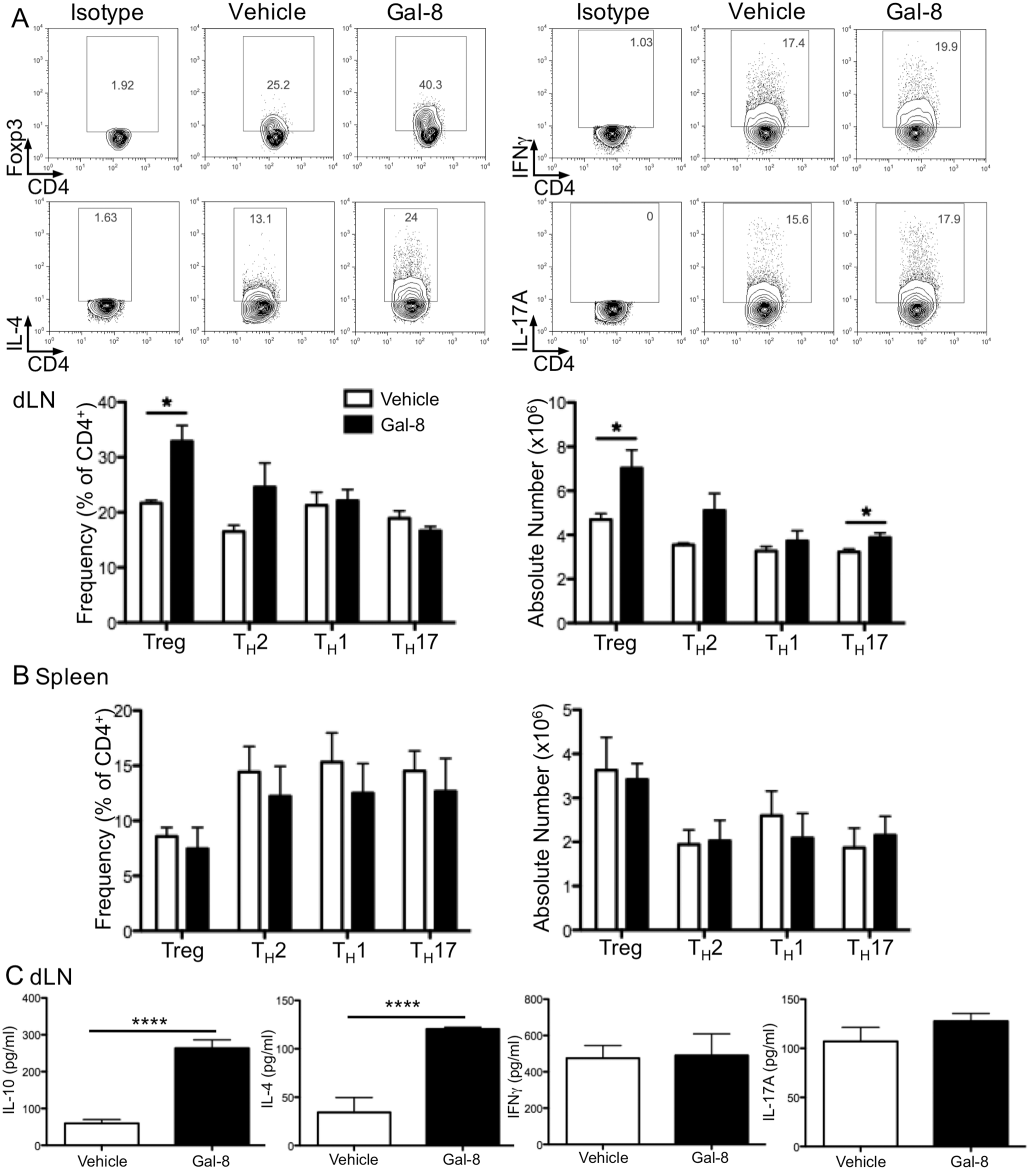
Galectin-8 promotes Treg cell differentiation in the dLN during EAU. dLN and spleen cells were collected from uveitic mice treated with Gal-8 or vehicle 24 d p.i. Cells from the dLN (A) or spleen (B) were stained for Foxp3, IL-4, IFNγ, and IL-17A, and representative scatter plots gated on CD4^+^ are shown, along with frequency and total number of each T cell subset. (C) Cytokine secretion from dLN cells stimulated *ex vivo* with IRBP_1-20_ for 48 h. FACS and ELISA samples were measured in triplicate. Error bars are mean ± SEM from three independent experiments with 18 vehicle- and 20 Gal-8-treated mice. P values were determined by Student’s *t* test. *, p < 0.05; ****, p < 0.0001.

During ocular inflammation, T cells are primarily activated in the cervical and submandibular dLNs. To determine whether Gal-8 ameliorates EAU pathology by influencing T cell differentiation, dLN from vehicle- and Gal-8-treated mice collected on day 24 p.i. were examined for the presence of T cell subsets by flow cytometry. Representative scatter plots gated on CD4^+^ cells are shown in Fig 3A. We found an increase in Treg cell frequency as well as number in the dLN of Gal-8-treated mice compared to vehicle-treated controls (Fig 3A). Although there was an increase in both the frequency and number of T_H_2 cells in the dLN of Gal-8-treated mice, this difference was not statistically significant. There was no difference in T_H_1 cells between the two groups (Fig 3A), but a 1.2-fold increase in total T_H_17 cells in the dLN of Gal-8-treated mice compared to the vehicle-treated control mice (Fig 3A). We observed no difference in frequency or number of CD4^+^ T cell populations in the spleen of Gal-8-treated mice as compared to vehicle-treated mice (Fig 3B). In addition, when dLN cells were restimulated *ex vivo* with IRBP_1-20_ for 2 days, and cytokines in the supernatant were measured by ELISA, cells from the dLN of Gal-8-treated mice secreted 4.4- and 3.5-fold more IL-10 and IL-4, respectively, than the cells from vehicle-treated mice, while IFNγ and IL-17A secretion were unaffected (Fig 3C).

**Fig 4:**
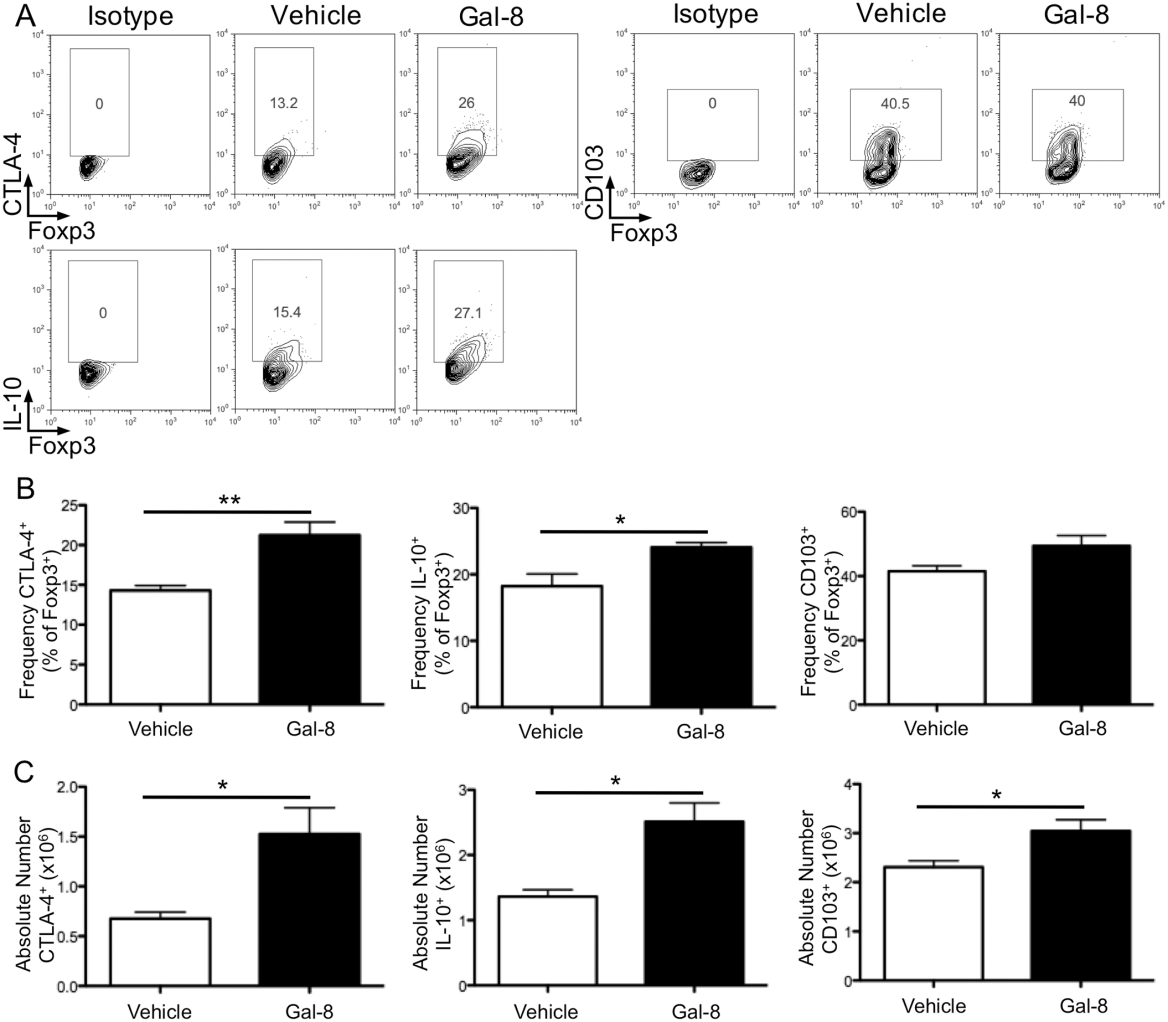
Galectin-8 promotes dLN Treg cell expression of suppressive molecules CTLA-4 and IL-10, and tissue-homing integrin CD103. Representative scatter plots (A), as well as frequency (B) and number (C) of CTLA-4, IL-10, and CD103 expressing Treg cells from the dLN of vehicle- and Gal-8-treated mice with uveitis. FACS samples were measured in triplicate. Error bars are mean ± SEM from three independent experiments with a total of 18 vehicle- and 20 Gal-8-treated mice. P values were determined by Student’s *t* test. *, p < 0.05; **, p < 0.01.

To assess the functional potential of Gal-8-induced Treg cells, dLN cells from vehicle- or Gal-8-treated mice were stained for CTLA-4 and IL-10, as well as for the tissue-homing integrin CD103, by flow cytometry. Scatter plots gated on CD4^+^Foxp3^+^ cells are shown in Fig 4A. The frequency (Fig 4B) and total number (Fig 4C) of CTLA-4^+^ and IL-10^+^ Treg cells was increased in the dLN of Gal-8-treated mice compared with control animals. Although there was no difference in the frequency (Fig 4B), there was a significantly higher number of CD103^+^ Treg cells in the dLN of Gal-8-treated mice (Fig 4C). Because CD103 is important for retention of Treg cells in the inflamed tissue [30], we next tested whether Treg cells would accumulate in the retina of Gal-8-treated mice, where CTLA-4 and IL-10 could inhibit activation and function of pathogenic T_H_1 and T_H_17 cells.

### Gal-8 treatment promotes a local anti-inflammatory response in the retina

**Fig 5:**
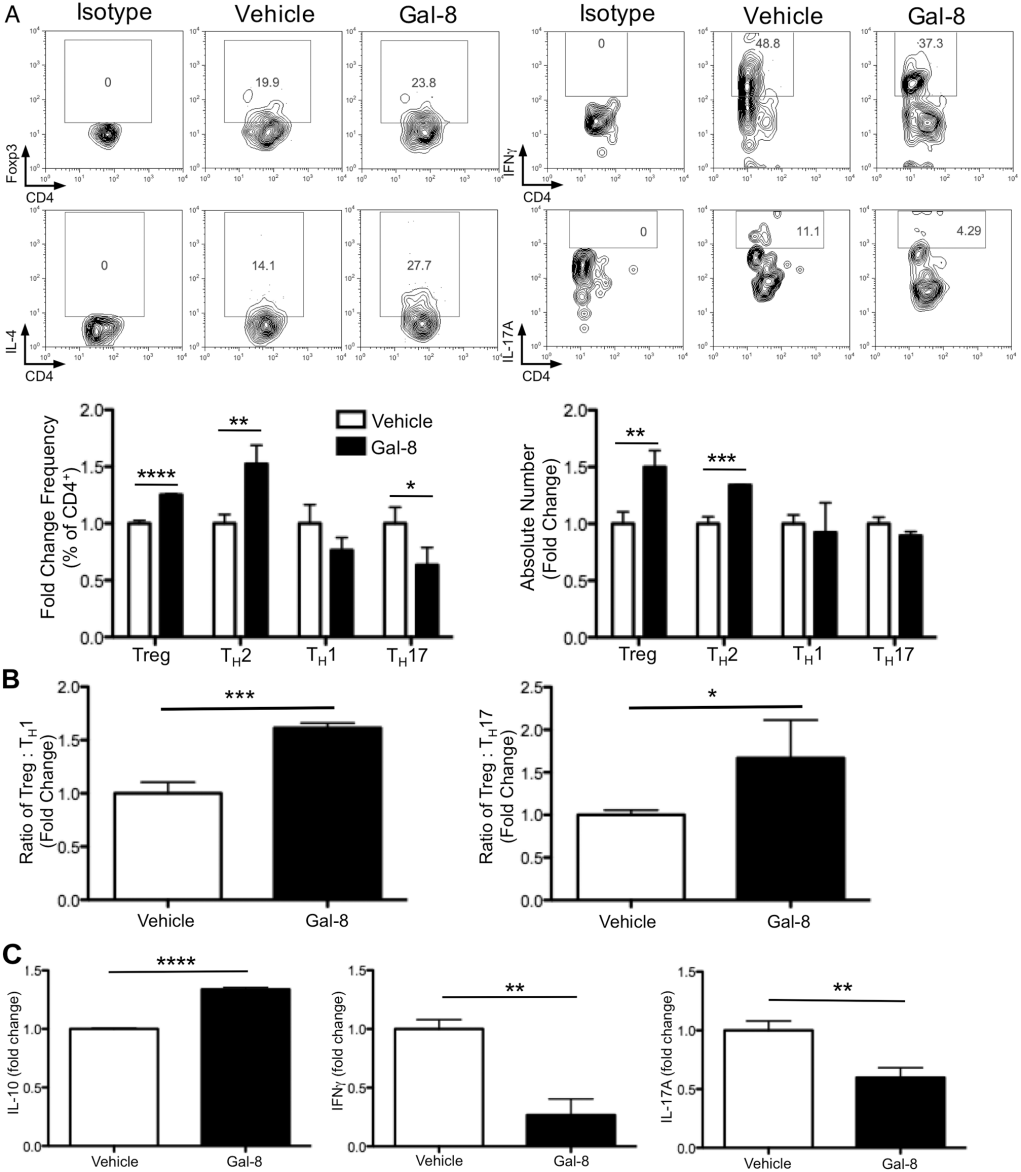
Galectin-8 inhibits inflammation of the retina. Retina-infiltrating cells were collected from uveitic mice treated with Gal-8 or vehicle at 24 d p.i. (A) Representative scatter plots of Foxp3, IL-4, IFNγ, and IL-17A staining are shown, and frequency and number of retinal Treg, T_H_2, T_H_1, and T_H_17 cells are quantified. (B) Cell ratios for total numbers of Treg per T_H_1 cell and Treg per T_H_17 cell in the retina. (C) Cytokines in pooled retinal extracts were measured by ELISA. (A-C) Retinas from five mice per group were pooled, and data are reported as fold change over control. Because of the limiting number of retinal lymphocytes, only one pooled FACS replicate was measured per group per experiment. ELISA samples were measured in quadruplicate. Error bars represent mean ± SEM of two independent experiments. P values were determined by Student’s *t* test. *, p < 0.05; **, p < 0.01; ***, p < 0.001; ****, p < 0.0001.

Retinal damage in EAU is mediated by cytokine production from T_H_1 and T_H_17 cells and can be inhibited by the action of Treg cells [6],[29]. To determine the effect of Gal-8 treatment on the local retinal immune environment, we analyzed T cell subsets in the retina of vehicle- and Gal-8-treated mice by flow cytometry (Fig 5A). Compared to the vehicle-treated control mice, there was a 25% increase in Treg cell frequency and a 50% increase in Treg number in the retina of Gal-8-treated mice (Fig 5A). The frequency of T_H_2 cells was 50% higher in Gal-8-treated versus vehicle-treated retinas, which resulted in a 34% increase in total retinal T_H_2 cells (Fig 5A). There was no significant difference in the number or frequency of T_H_1 cells in the retinas of Gal-8-treated mice, but the frequency of T_H_17 cells was reduced by 37% compared to vehicle-treated mice (Fig 5A). The ratio of Treg: T_H_1 cells was increased 1.6-fold and the ratio of Treg: T_H_17 cells was increased 1.7-fold in the retinas of Gal-8-treated mice compared to vehicle treatment (Fig 5B). The anti-inflammatory cytokine IL-10 was increased by 34% in retinal extracts from the Gal-8-treated group compared to the vehicle-treated control group (Fig 5C). In contrast, there was a dramatic decrease in soluble retinal IFNγ and IL-17A in Gal-8-treated mice (Fig 5C), consistent with the reduced pathology of these mice.

**Fig 6:**
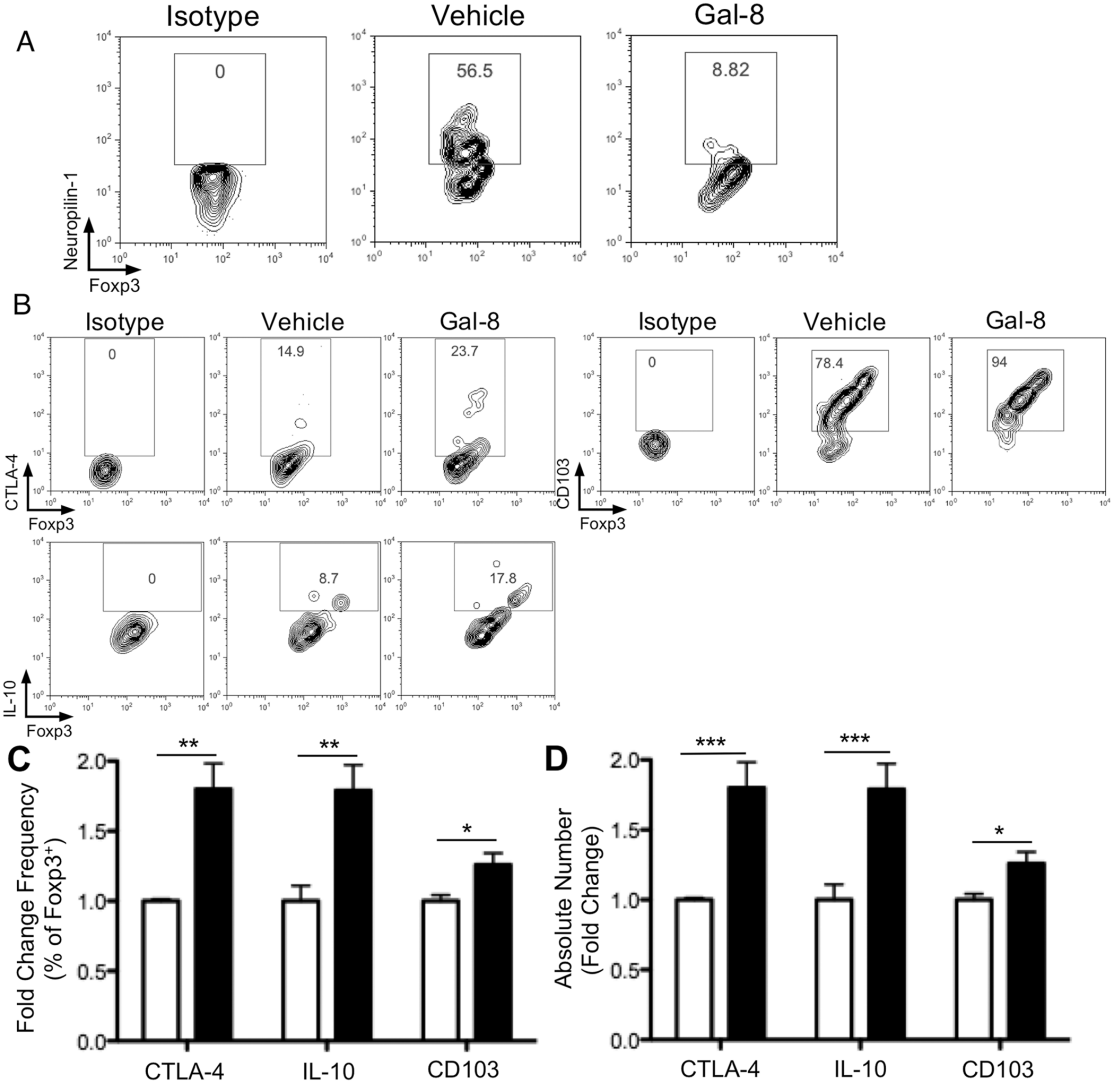
Treg cells in Gal-8-treated retinas are highly anti-inflammatory, inducible Treg cells. Retina-infiltrating cells were collected from uveitic mice treated with Gal-8 or vehicle at 24 d p.i. (A) Representative scatter plots show neuropilin-1 staining of Foxp3^+^ Treg cells. (B-D) Representative scatter plots (B), as well as frequency (C) and number (D) of CTLA-4, IL-10, and CD103 positive retinal Treg cells. (B-D) Retinas from five mice per group were pooled, and data are reported as fold change over control. Because of the limiting number of retinal lymphocytes, only one pooled FACS replicate was measured per group per experiment. Error bars represent mean ± SEM of two independent experiments. P values were determined by Student’s *t* test. *, p < 0.05; **, p < 0.01; ***, p < 0.001.

Neuropilin-1 is specifically expressed by Treg cells that originate in the thymus, but is not expressed on Treg cells induced in the periphery [31]. In the retina of vehicle-treated mice, 56% of Treg cells expressed neuropilin-1, whereas only 9% of retina-infiltrating Treg cells in Gal-8-treated mice expressed neuropilin-1. This suggests that 90% of Treg cells in the Gal-8-treated retinas are of extra-thymic origin (Fig 6A). Consistent with our observations in the dLN (Fig 4), a higher percentage and number of retinal Treg cells from Gal-8-treated mice expressed CTLA-4, IL-10, and CD103 (Fig 6B–6D). Taken together, these data suggest that Gal-8 treatment modulates the severity of EAU pathology by enhancing anti-inflammatory Treg cell responses.

## Discussion

Autoimmune uveitis is a potentially blinding disease associated with T_H_1 and T_H_17 cytokine production and ameliorated by Treg cells. Despite rodent models of EAU that reproduce aspects of the human disease, development of rational treatments for uveitis has been slow. To the best of our knowledge, this is the first report demonstrating that Gal-8, a carbohydrate-binding protein, ameliorates EAU pathology. Gal-8 treatment in the mouse model of EAU: (i) resulted in increased frequency and number of CTLA-4^+^, IL-10^+^, and CD103^+^ Treg cells in the dLN and retina, and (ii) downregulated the production of proinflammatory cytokines, IFNγ and IL-17A, and upregulated the levels of the anti-inflammatory cytokine IL-10, in the retina. Taken together, these results indicate that Gal-8 functions as an anti-inflammatory protein and could serve as a valuable therapeutic intervention not only for uveitis, but also for a broad range of autoimmune and chronic inflammatory conditions.

In the EAU model, Gal-8 also promoted development of T_H_2 cells, which has been associated with less severe EAU pathology. Strains of mice with an inherent T_H_2 bias are generally resistant to induction of EAU [32]. Others have demonstrated that skewing the T cell response away from T_H_1 and T_H_17 to T_H_2 and Treg significantly ameliorates EAU pathology [33],[34], although the precise mechanism by which T_H_2 cells inhibit EAU is still poorly understood. Additional studies are needed to directly ascertain the relative importance of the increased T_H_2-type response evoked in the Gal-8 treated mice in the current study. The role of CD4^+^ effector T cells, particularly T_H_1 and T_H_17 cells is well documented in the pathogenesis of EAU. Because either T_H_1 or T_H_17 cells can drive immunopathology in EAU [29], an effective therapy should ideally target both T_H_1 and T_H_17 cells. In this regard, our finding that treatment with Gal-8 during the course of uveitis markedly decreases the soluble levels of both the T_H_1 cytokine, IFNγ, as well as the T_H_17 cytokine, IL-17A, in the retina is of interest. Likewise, our finding that Gal-8 treatment in the EAU model enhances soluble IL-10 in the retina is of potential therapeutic value. IL-10 is thought to play a critical role in the inhibition of EAU pathology. IL-10 mRNA expression was shown to increase during the natural resolution of rat EAU [35]. Moreover, treatment with recombinant IL-10 inhibits EAU, whereas administration of an anti-IL-10 Ab significantly exacerbates pathology [36]. We observed that Gal-8 treatment i.p. affects T cell differentiation at sites of active inflammation, such as the dLN and retina, but does not systemically alter the differentiation state of naïve T cells, as we observed no change in CD4^+^ T cell subpopulation frequency in the spleen. This is consistent with the emerging role of galectins in restoration of immune homeostasis [7],[18],[21].

The data in the present study do not exclude the possibility that Gal-8 may have additional effects on other immune cell types to decrease EAU pathology, however the role of Gal-8 in the function of most immune cells is still poorly understood. Lupus patients often have high titers of anti-Gal-8 serum autoAbs [37], suggesting that Gal-8 plays an anti-inflammatory role in an autoimmune disease with little T cell involvement. Gal-8 has been shown to promote neutrophil adhesion and superoxide production via the C-terminal CRD [38], as well as plasma cell differentiation and antibody production [39]. Additionally, Gal-8 is expressed in the thymus and can induce apoptosis of thymocytes, which has been postulated to play a role in tolerance [40]. Although it is possible that Gal-8 may affect development of Treg cells in the thymus (nTreg cells), our finding that the majority of Treg cells in Gal-8-treated retinas are peripherally-induced iTreg cells suggests that Gal-8 either promotes the differentiation or recruitment of iTreg cells.

Others have demonstrated that Gal-1 and Gal-9 positively regulate Treg cell function [11],[12],[21], whereas Gal-3 negatively regulates Treg cell expansion in the context of autoimmunity [41]. In this respect, in a preliminary study, we have observed that Gal-8 promotes Ag-specific and polyclonal differentiation of Treg cells *in vitro* (Sampson and Panjwani, unpublished observations). Although most members of the galectin family bind galactose-containing residues, variability in the CRD among galectins results in unique fine specificities for more complex galactose-containing oligosaccharides [42]. As a result, each galectin may have profoundly different ligand preference based on glycosylation patterns, with consequent specific downstream effects [19],[43],[44]. In this respect, the carbohydrate-binding affinity of the N-terminal CRD of Gal-8 is unique among members of galectin family in that it has a high affinity for 3’- sialylated glycoconjugates [45]. This unique carbohydrate specificity and structure could make Gal-8 a selective drug target.

Finally, our finding that Gal-8 treatment ameliorates EAU pathology has broad implications for developing novel therapeutic strategies not only for uveitis but also for a wide array of autoimmune diseases including multiple sclerosis, ulcerative colitis, and atherosclerosis, which are mediated by T_H_1 and T_H_17 cytokines and ameliorated by the action of Treg cells [41],[46],[47]. The most common treatment for uveitis and other autoimmune disorders has not changed in decades. Therapy is based on nonspecific immunosuppression, such as corticosteroids and TNFα blocking agents, despite the fact that these treatments are well known to be associated with severe side effects and toxicity, leaving patients immunocompromised and susceptible to opportunistic infection or reactivation of latent tuberculosis [48]. Thus, there is a clear unmet need for developing more specific treatments based on increased understanding of basic disease mechanisms. It is our hope that Gal-8-based therapies could serve to ameliorate the clinical outcome of human uveitis and other T_H_1- and T_H_17-mediated autoimmune pathologies.
